# New Insights on the Relationship between Leptin, Ghrelin, and Leptin/Ghrelin Ratio Enforced by Body Mass Index in Obesity and Diabetes

**DOI:** 10.3390/biomedicines9111657

**Published:** 2021-11-10

**Authors:** Adela-Viviana Sitar-Tǎut, Angela Cozma, Adriana Fodor, Sorina-Cezara Coste, Olga Hilda Orasan, Vasile Negrean, Dana Pop, Dan-Andrei Sitar-Tǎut

**Affiliations:** 1 Internal Medicine Department, 4th Medical Clinic, Faculty of Medicine, “Iuliu Hațieganu” University of Medicine and Pharmacy, 400012 Cluj-Napoca, Romania; angelacozma@yahoo.com (A.C.); secara.sorina@yahoo.com (S.-C.C.); olgaorasan@yahoo.com (O.H.O.); vasile.negrean@umfcluj.ro (V.N.); 2 Clinical Center of Diabetes, Nutrition, Metabolic Diseases, Faculty of Medicine, “Iuliu Hațieganu” University of Medicine and Pharmacy, 400012 Cluj-Napoca, Romania; adifodor@yahoo.com; 3 Department of Cardiology, Clinical Rehabilitation Hospital, Faculty of Medicine, “Iuliu Hațieganu” University of Medicine and Pharmacy, 400012 Cluj-Napoca, Romania; pop67dana@gmail.com; 4 Business Information Systems Department, Faculty of Economics and Business Administration 58-60 Theodor Mihaly Street, “Babeş-Bolyai” University, 400591 Cluj-Napoca, Romania; dan.sitar@econ.ubbcluj.ro

**Keywords:** obesity, body mass index, diabetes, leptin, ghrelin

## Abstract

Currently, adipose tissue is considered an endocrine organ, however, there are still many questions regarding the roles of adipokines—leptin and ghrelin being two adipokines. The purpose of the study was to assess the relationship between the adipokines and their ratio with obesity and diabetes. Methods: Sixty patients (mean age 61.88 ± 10.08) were evaluated. Cardiovascular risk factors, leptin, ghrelin, and insulin resistance score values were assessed. The patients were classified according to their body mass index (BMI) as normal weight, overweight, and obese. Results: 20% normal weight, 51.7% overweight, 28.3% obese, and 23.3% diabetic. Obese patients had higher leptin values (in obese 34,360 pg/mL vs. overweight 18,000 pg/mL vs. normal weight 14,350 pg/mL, *p* = 0.0049) and leptin/ghrelin ratio (1055 ± 641 vs. 771.36 ± 921 vs. 370.7 ± 257, *p* = 0.0228). Stratifying the analyses according to the presence of obesity and patients’ gender, differences were found for leptin (*p* = 0.0020 in women, *p* = 0.0055 in men) and leptin/ghrelin ratio (*p* = 0.048 in women, *p* = 0.004 in men). Mean leptin/BMI and leptin/ghrelin/BMI ratios were significantly higher, and the ghrelin/BMI ratio was significantly lower in obese and diabetic patients. In conclusion, obesity and diabetes are associated with changes not only in the total amount but also in the level of adipokines/kg/m^2^. Changes appear even in overweight subjects, offering a basis for early intervention in diabetic and obese patients.

## 1. Introduction

It is well known that in last 50 years diet (with an excessive supply of energy delivered with food), lower energy expenditure and lifestyle changes are responsible for increasing prevalence of obesity (2.5 billion adults being reported as overweight or obese in 2016 [1]) and diabetes mellitus (more than 400 million adults diagnosed in 2019) [2,3,4]. Obesity and diabetes are both considered at this time public health issues [5,6,7,8,9]. Hundreds of millions of people, all over the world [4,10,11], are confronting their effects, literature suggesting a strong association between them [12]. 

Currently, obesity is considered a heterogeneous syndrome [13], the same fat mass excess being associated with various types of metabolic profile and risk [3,14]. Various types of intervention for obesity prevention and treatment have been proposed (diets, pharmacological interventions, or bariatric surgery). It is very important to make an accurate selection of obese patients gaining the most benefits, but also identify those with developing high-risk complications.

Taking into consideration the previously mentioned data, in recent years, the focus has shifted from adipose tissue as a fat storage organ [15] to an endocrine and immune organ [7,10,16,17,18,19,20,21,22,23], secreting various types of molecules [7,11,15,22]. The last decade has witnessed an increase in the number of discovered adipokines, with more than 600 adipokines being secreted by adipose tissue, and with an increasing need to identify their roles and clinical relevance [18]. Adipokines are involved in appetite regulation [22], energy balance, glucose homeostasis, lipid metabolism, in the pathogenesis of insulin resistance [18,20], diabetes mellitus, atherosclerosis, hypertension, metabolic syndrome, cardiovascular disease, and cancer [21,24,25,26,27]. Currently, inadequate adipokines’ secretion can emphasize adipose tissue dysfunction [15,18,21], linking obesity to other comorbidities (including diabetes) [15,18,19,20,22,23,28,29].

Despite this great interest in the implied mechanism in obesity and diabetes, there are still many questions in the debate on the role of adipokines. Most research on this topic has focused on the individual adipokines’ roles and values in diabetes or obesity. To the best of our knowledge, their relationship and the influence of body mass index over them have rarely been evaluated. 

Knowledge of the possible interactions and pathological implications is needed for personalized prevention [18], early diagnosis, estimation of the risk of complications, and early intervention to reduce morbidity and mortality.

Leptin and ghrelin appear to be involved in glucose and lipid metabolism, eating behavior [7,11,22,30] and energy balance [1,23], playing important roles in hormonal regulation of food intake [17,30], being potent appetite influencers in the opposite direction [1,7,26]. Due to their interaction, they are considered in a “ghrelin-leptin tango” [17]. 

The aim of this work is to extend our knowledge on the relationship between adipokine (leptin, ghrelin) and their ratio enforced by body mass index, obesity, diabetes, and metabolic syndrome. Moreover, we have intended to evaluate a possible subtle relationship between adipokines and body mass index (their ratio)—a possible substrate for framing the same BMI patient category in different risk classes.

## 2. Materials and Methods

The current study was conducted in the Department of Cardiology of the Rehabilitation Hospital in Cluj-Napoca, a total number of 60 consecutively recruited hospital-admitted patients (44 women) were enrolled in this study. The mean age was 61.88 ± 10.08 years. Subjects who did not consent in writing to participate were excluded from the present study; also, those who present systemic or inflammatory diseases. At the same time, taking into account recently published papers with controversial data on the interaction between lipid-lowering therapy (depending on dose, duration, and type of treatment) and adipokines levels [31,32,33,34,35,36,37], patients with no data related to this topic have also been excluded.

A complete clinical examination was performed by a physician (according to the current European Society of Cardiology guidelines). Bodyweight, height, body mass index (BMI, calculated as weight divided by squared height, expressed as kg/m^2^), waist circumference (in centimeters), present or past smoking, obesity, presence of dyslipidemia (total cholesterol ≥ 200 mg/dL or serum triglycerides ≥ 150 mg/dL), hypertension (blood pressure ≥ 140/90 mmHg or under hypotensive treatment), and diabetes were recorded.

For each patient, a blood sample was collected in the morning (between 7:00 a.m. and 9:00 a.m.); lipid fractions, glycemia were determined. The insulin resistance score was assessed as homeostatic model assessment (HOMA index) = insulin (μU/mL) × glycemia (mg/dL)/405. 

Using the commercially available ELISA kits method (enzyme-linked immunosorbent assay, R&D Systems Inc., Minneapolis, MN, USA) serum total ghrelin (pg/mL) and serum leptin (pg/mL) levels were determined for each patient.

Patients were classified according to their body mass index in normal weight (body mass index BMI 18.5–24.9 kg/m^2^), overweight (BMI 25–29.9 kg/m^2^), and obese (BMI ≥ 30 kg/m^2^). The classification of metabolic syndrome (MetS) was based on International Diabetes Federation (IDF) guidelines (central obesity plus any two of the following: triglycerides ≥ 150 mg/dL, low HDL-cholesterol, increased blood pressure, elevated fasting plasma glucose, or diabetes) [38].

The local University Ethics Committee (following the Declaration of Helsinki) approved the study protocol. 

The statistical packages MedCalc version 10.3.0.0 (MedCalc Software, Ostend, Belgium) and SPSS for Windows version 16.0 (IBM Corporation, Armonk, New York, NY, USA) were used for processing statistical analysis. For all quantitative variables, distribution’s normality was tested using the Kolmogorov–Smirnov and D’Agostino–Pearson tests; quantitative data were presented as the mean ± standard deviation, median values, respectively; qualitative data as numbers and percentages. Independent sample *t*-test, Mann–Whitney, χ2 test, ANOVA (analysis of variance), or Kruskal–Wallis test were used to analyze differences between variables or groups; relationships were assessed using Spearman and Pearson correlation coefficients. Univariate and multivariate regression were used to identify independent prognostic factors. A *p*-value < 0.05 was considered statistically significant. 

## 3. Results

Twelve (20%) patients presented as normal weight, 31 (51.7%) overweight and 17 (28.3%) were obese; 23.3% were diabetics (type 2 DM—all of them), 47 (78.3%) hypertensive, 11 (18.3%) current smokers, 41 (68.3%) with dyslipidemia, and 71.7% with MetS. Moreover, 53.3% were diagnosed with cardiovascular disease (ischemic heart disease, heart failure, peripheral artery disease, previous stroke). 

The mean age of the evaluated patients was 61.88 ± 10.08; no noteworthy differences (regarding age) were found between the groups (normal weight vs. overweight vs. obese ones). The tests revealed significant differences between the three groups in relationship with abdominal circumference (*p* < 0.001), body mass index (*p* < 0.001), glycemia (*p* = 0.016), presence of diabetes (*p* = 0.0099). 

The overall mean ± SD (median) values were for ghrelin—39.55 ± 18.90 (34.25) pg/mL, for HOMA-index—2.07 ± 1.19 (1.72), and for leptin/ghrelin ratio—771.68 ± 791.43 (508.61). The characteristics of the studied group are presented extensively in Table 1. 

**Figure 1:**
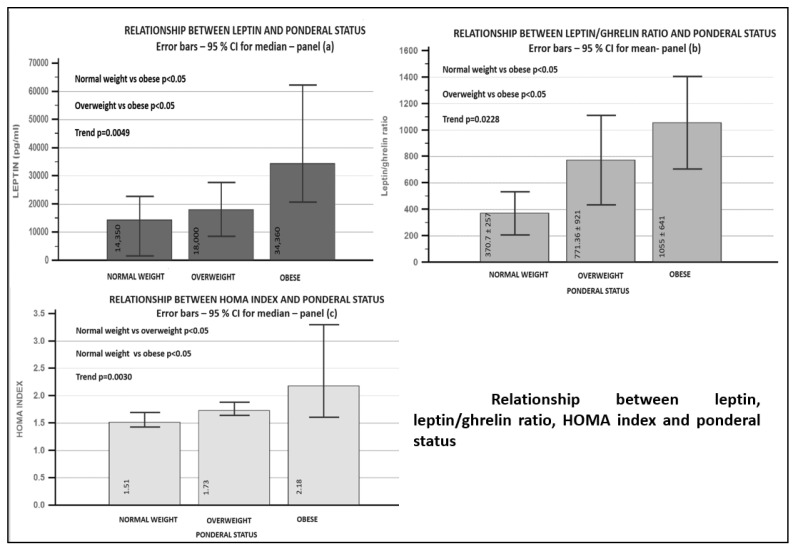
Relationship between adipokines, insulin resistance score and ponderal status. Panel (**a**)—relationship between leptin and ponderal status; panel (**b**)—relationship between leptin/ghrelin ratio and ponderal status; panel (**c**)—relationship between HOMA index and ponderal status.

When absolute values were compared, obese patients presented higher values of leptin (*p* trend = 0.0049), leptin/ghrelin ratio (*p* trend = 0.0228) and HOMA index (*p* trend = 0.003)—complete data are presented in Figure 1. Significant differences were found between obese patients and overweight (for leptin *p* < 0.05, for leptin/ghrelin ratio *p* < 0.05) and between obese and normal weight patients (for leptin *p* < 0.05, for leptin/ghrelin ratio *p* < 0.05). No relationship was found between ghrelin level and ponderal status (*p* = NS).

Stratifying the analyzes according to the presence of obesity and patients’ gender, significant differences were found for leptin in both sexes. Obese female presented greater values (16,930 pg/mL in normal weight vs. 32,227 pg/mL in overweight vs. 39,270 pg/mL in obese, *p* trend = 0.0020); same results were found for men (1560 pg/mL in normal weight vs. 3480 pg/mL in overweight vs. 20,240 pg/mL in obese, *p* trend = 0.0055). 

For the leptin/ghrelin ratio, significant differences were found between groups for both sexes—for women (437.93 ± 225.87 vs. 1019.48 ± 992.11 vs. 1295.81 ± 669, *p* trend = 0.048) and for men (34.55 ± 4.29 vs. 164.84 ± 140.84 vs. 478.11 ± 189.65, *p* trend = 0.004). 

HOMA index was significantly higher in obese women (*p* trend = 0.008), but not in obese men (*p* trend = 0.11). 

**Figure 2:**
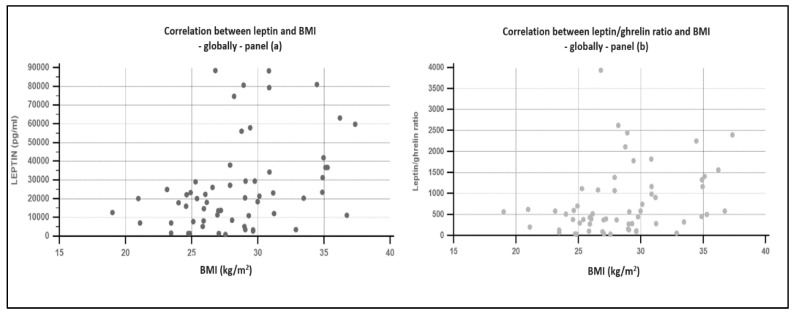
Correlation between leptin, leptin/ghrelin ratio, and body mass index. Panel (**a**)—correlation between leptin value and body mass index; panel (**b**)—correlation between leptin/ghrelin ratio and body mass index.

Globally, significant correlations were found between leptin and BMI (rho = 0.402, *p* = 0.001), insulin (rho = 0.271, *p* = 0.036), ponderal status (rho = 0.420, *p* = 0.0012) and patients’ sex (rho = −0.57, *p* < 0.001). Significant relationships were found between ghrelin and age (rho = −0.344, *p* = 0.007), diabetes presence (rho = −0.266, *p* = 0.04). The leptin/ghrelin ratio correlated with BMI (r = 0.304, *p* = 0.018), ponderal status (r = 0.29, *p* = 0.021), diabetes presence (r = 0.318, *p* = 0.013), insulin (r = 0.287, *p* = 0.026), and patients’ sex (r = −0.404, *p* = 0.001). Data are presented in Figure 2. No associations were found between leptin, ghrelin, or leptin/ghrelin ratio and glycemia, HOMA index, lipid fractions, abdominal circumference, systolic or diastolic blood pressure. 

In women, leptin correlates with weight (rho = 0.496, *p* = 0.001), BMI (rho = 0.577, *p* < 0.001), abdominal circumference (rho = 0.505, *p* < 0.001), diabetes (rho = 0.408, *p* = 0.006) and ponderal status (rho = 0.537, *p* < 0.001) ghrelin with age (rho = −0.434, *p* = 0.003), weight (rho = 0.304 *p* = 0.004), and the leptin/ghrelin ratio with age (r = 0.363, *p* = 0.015), BMI (r = 0.387, *p* = 0.009), abdominal circumference (r = 0.338, *p* = 0.018), diabetes (r = 0.477, *p* = 0.001), and ponderal status (r = 0.359, *p* = 0.017). In men, leptin correlates with age (rho = −0.510, *p* = 0.043), weight (rho = 0.576, *p* = 0.02), BMI (rho = 0.697, *p* = 0.025), ponderal status (rho = 0.819, *p* < 0.001), and the leptin/ghrelin ratio with BMI (r = 0.603, *p* = 0.013) and ponderal status (r = 0.735, *p* = 0.001)

The predictors of leptin, ghrelin, and the leptin/ghrelin ratio were studied using univariate and multivariate analysis. For leptin in the univariate analysis, independent predictors were body mass index (R^2^ = 0.125, *p* = 0.003), insulin (R^2^ = 0.128, *p* = 0.005), ponderal status (R^2^ = 0.150, *p* = 0.002), and patient sex (R^2^ = 0.210, *p* < 0.001). In the multivariate analysis (stepwise method), independent factors were ponderal status and patient sex. 

In the univariate analysis, for ghrelin age (R^2^ = 0.166, *p* = 0.001) and for the leptin/ghrelin ratio BMI (R^2^ = 0.093, *p* = 0.018), ponderal status (R^2^ = 0.088, *p* = 0.021), diabetes (R^2^ = 0.101, *p* = 0.013), insulin (R^2^ = 0.082, *p* = 0.026), and patient sex (R^2^ = 0.163, *p* = 0.001) were independent predictive factors. In the multivariate analysis, for the leptin/ghrelin ratio, patients ‘gender, diabetes, and body mass index were independent factors.

No significant differences were found in the values of leptin and ghrelin between patients with MetS vs. those without MetS. Patients with MetS presented higher values of leptin/ghrelin (869.19 ± 845 vs. 525.03 ± 584, *p* = 0.07) and the HOMA index (1.83 vs. 1.57, *p* = 0.004). 

Considering diabetic patients, as highlighted in Table 2, globally significant differences were found regarding insulin, HOMA index, ghrelin (*p* = 0.0409), and the leptin/ghrelin ratio (*p* = 0.0131). Differences were also present in women, in men registered *p* being non-significant.

**Figure 3:**
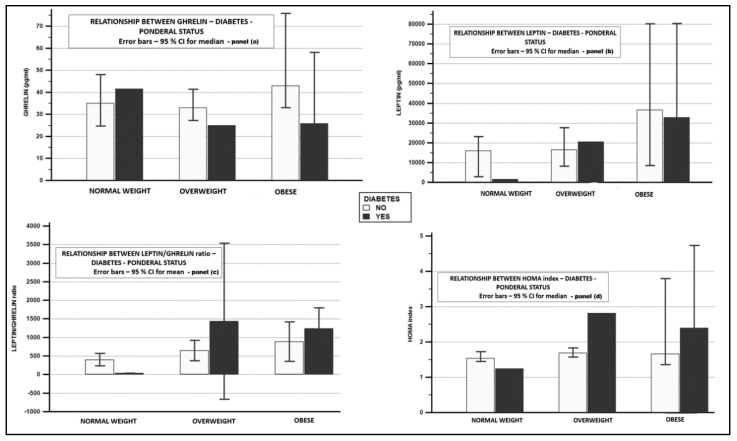
Relationship between adipokines—obesity—diabetes. Panel (**a**)—relationship between ghrelin—diabetes—ponderal status; panel (**b**)—relationship between leptin—diabetes—ponderal status; panel (**c**)—relationship between leptin/ghrelin ratio—diabetes—ponderal status; panel (**d**)—relationship between HOMA index—diabetes—ponderal status.

Diabetic and obese patients (vs diabetic and nonobese patients) presented greater leptin values (32,810 pg/mL vs. 14,505 pg/mL), lower ghrelin levels (25.75 pg/mL vs. 29 pg/mL); no difference in relationship with the leptin/ghrelin ratio (1242.42 ± 663 pg/mL vs. 1201.10 ± 1616 pg/mL, *p* = NS) was found. Detailed data regarding the relationship between BMI category and diabetes are presented in Figure 3.

**Figure 4:**
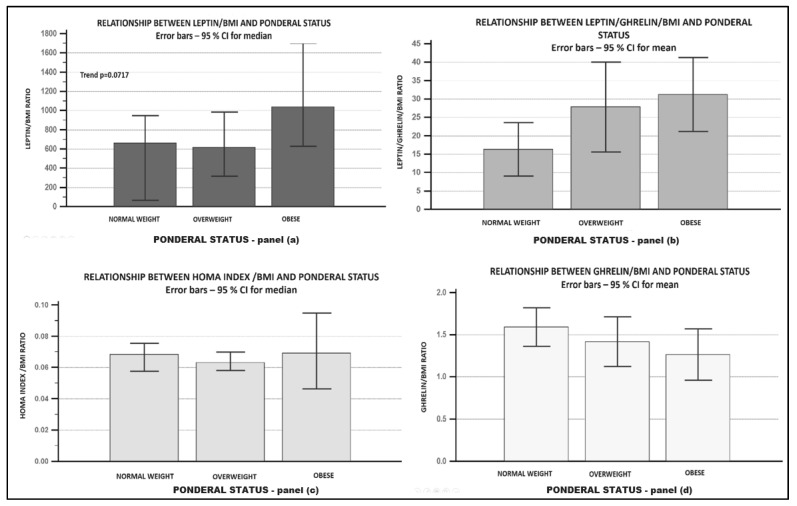
Relationship between leptin/BMI ratio, leptin/ghrelin/BMI ratio, Ghrelin/BMI ratio, HOMA index/BMI ratio and ponderal status. Panel (**a**)—relationship between leptin/BMI ratio—ponderal status; panel (**b**)—relationship between leptin/ghrelin/BMI ratio—ponderal status; panel (**c**)—relationship between HOMA index/BMI ratio—ponderal status; panel (**d**)—relationship between ghrelin/BMI ratio—ponderal status.

After calculating the adipokines/BMI ratio (data presented in Table 3), we should mention that no statistical significance was achieved, mean ghrelin/BMI was the lowest in obese subjects (1.59 ± 0.35 in normal weight vs. 1.41 ± 0.8 in overweight vs. 1.26 ± 0.58 in obese). The mean leptin/BMI ratio and leptin/ghrelin/BMI ratio were highest in obese patients (data in Table 3 and graphic representation in Figure 4). 

In metabolic syndrome, respectively, in diabetic patients, a lower ghrelin/BMI ratio and a higher leptin/ghrelin/BMI ratio were also found. 

Globally, the determined area under the ROC curve for MetS identification was 0.687 (Se = 44.2%, Sp = 82.4%, criterion > 600.54) for the leptin/ghrelin ratio. For the HOMA index, the AUROC was 0.740 (Se = 48.8%, Sp = 100%, criterion > 1.83). 

In men, the leptin/ghrelin ratio had a better capacity to identify patients with metabolic syndrome (AUROC = 0.923, Se = 76.9%, Sp = 100%) compared to leptin (AUROC = 0.821), ghrelin (AUROC = 0.718), or the HOMA index (AUROC = 0.654); *p* = 0.09 between the leptin/ghrelin ratio AUROC vs. AUROC-HOMA. 

In women, no significant differences were found between AUROCs (AUROC-HOMA = 0.752 vs. AUROC-leptin = 0.706 vs. AUROC-ghrelin = 0.536 vs. AUROC-leptin/ghrelin ratio = 0.690). Data are presented in Table 4. 

The presence of diabetes was better identified by the HOMA index (AUROC leptin/ghrelin ratio = 0.658, AUROC leptin = 0.632, AUROC ghrelin = 0.682, AUROC HOMA index = 0.831); the results were similar in both sexes. 

## 4. Discussion

The increase in obesity and diabetes prevalence has important consequences on population health, the financial burden on the health system [18], and the impact on all body systems [3,7,8,39,40,41,42,43]. 

The body mass index (BMI) represents the most used tool to assess the degree of obesity. Although early studies believed that it is all about increasing in size and number of adipocytes, recent studies pointed to metabolism dysregulation, insulin resistance, systemic inflammation [18,44], responsible being the adipokines, cytokines, extracellular matrix proteins, vasoactive substances, and the release of hormone-like action proteins [7,45,46,47]. New data have suggested the idea that this variability in adipose tissue composition, distribution, and substance release is a substrate for people in the same BMI category being framed in various risk levels [3,7,13,21], and a key factor in obesity-related metabolic disorders [12]. Substances secreted by dysfunctional adiposity have pro-inflammatory, pro-thrombotic, and pro-atherogenic effects, but also affect vascular tone and motricity, endothelial function [7], promoting cardiac fibrosis appearance [7]. 

On the other hand, it is well known that a large proportion of type 2 diabetics are obese and, inversely, type 2 diabetes is more frequently met in obese people [21,48], a clear connection between those being already established. Over the last 10 years, the focus has shifted from two separate entities (obesity and diabetes) to an interwoven perspective. 

Despite great interest in a complex relationship between adipokines–obesity–diabetics, many aspects are still unclear. Today, many theories and techniques have evolved to understand and prevent, to highlight the already appeared related complication of type 2 diabetes and obesity [49,50,51,52], to create estimative risk models [53]. 

On the other hand, the underlined mechanisms are not, at this moment, fully explained, and adipokines secretion dysregulation is considered as a possible missing chain between two entities. 

Leptin and ghrelin are the main hormones that, working together, but in an opposite manner [26,27], regulating reciprocally [54], influence appetite and hunger sensations [17,26]. 

Leptin (from the Greek word leptos, which means thin [11]) is secreted by adipose tissue (in proportion with fat stores [29,55]), but also by the stomach and mammary gland [29]. It influences dietary intake, regulates food intake [7,15,17,22,23,29,55], energy consumption [15,29], induces the satiety sensation—“a satiety hormone” [21,22,23,26,47], and, consequently, determines the number of adipose deposits [25,56]. At the same time, it is considered a pro-inflammatory adipokine [15], being involved in low inflammation associated with an increased amount of fat tissue [22]. Most forms of obesity are associated even with leptin resistance [15,23]. Different mechanisms are responsible, including the fact that chronic high leptin level leads to leptin insensitivity [57]. Our results are in line with previous ones [11,17,18,22,28,55,58,59,60,61,62], showing higher levels in obese or diabetic patients and a positive correlation with body mass index (as reported in [15,28]). 

Ghrelin, a stomach-derived hormone [30] secreted by P/D1 cells [17,63] also has an important role in short-term appetite regulation [11,17,30,64,65] and stimulation [11,26,27,60,63,66,67], but also involved in lipogenesis [67], insulin sensitivity, having anti-inflammatory properties [65], blocking the renin-angiotensin system [7,67], decreasing sympathetic activity [67,68], influencing blood pressure and heart rate [67,68], and finally being involved in cardiovascular disease development (low values being associated with increase global cardiovascular risk [54,69,70]). Previously reported data suggested the idea that a low ghrelin level could be one of the pathogenetic pathways of type 2 diabetes development [70,71]. 

The results presented in the current study are not in line with those previously published by [17,64,72,73,74,75], who found low ghrelin levels in obese patients, but in accordance with a 2021 published study finding no significant differences between obese and normal-weight patients regarding ghrelin levels [1]. We do not have a clear explanation for these discrepancies; probably, ghrelin levels are elevated due to 12 h fasting or food restriction [11,26,63], starvation [63]—knowing that its concentration increases before meal intake [17,66] and is influenced by low meal frequency, diet composition, exercise, and lifestyle [66]. There have also been published studies reporting the nocturnal increase in ghrelin levels [76,77]. At the same time, the literature describes the “obesity paradox”—obese subjects appear to have heterogeneous phenotypes [13]—from Metabolically UnHealthy Obesity (MuHOB) to Metabolically Obese Normal Weight (MONW or metabolically unhealthy normal BMI—normal BMI associated with obesity-related metabolic complications—more than 20% in US adults [3]) and Metabolically Healthy Obesity (MHOB—10–30% in European obese, more frequently met in women [13], 10% of US adults [3]). Just the simple use of the body mass index does not allow us to accurately discriminate between lean and fat mass, between MuHOB-MONW-MHOB [3,13]. 

However, our results support other published theories [65,70,71,78], theories founding lower ghrelin levels in diabetic patients. 

Due to the discrepancy between the previous results, new parameters have been evaluated such as the leptin/ghrelin ratio, leptin/BMI ratio, ghrelin/BMI ratio, and leptin/ghrelin/BMI ratio. 

The leptin/ghrelin ratio appears to be a hunger regulator [17], a higher ratio being associated with hunger and decreased appetite [17]. The previous hypothesis enunciated suggested the fact that leptin/ghrelin ratio can be used to identify subjects with an unfavorable evolution after obesity weight-loss therapeutic treatment [54,79], with weight regain after successful weight loss [54]. To our best knowledge, only a few studies have explored its relationship with obesity—metabolic syndrome—diabetes. Our results reinforce the data reported by [17,26,54], the leptin/ghrelin ratio being significantly higher in overweight/obese patients or diabetic or metabolic syndrome patients. 

Furthermore, we should mention the fact that, in men, the leptin/ghrelin ratio had a very good discriminatory capacity for metabolic syndrome (AUROC = 0.923). Compared to previous studies that evaluated other parameters (such as the leptin/adiponectin ratio, HOMA index, QUICKI index, McAuley index, triglycerides/HDL-cholesterol ratio, cholesterol/HDL-cholesterol ratio, different measurement in abdominal CT—[80,81,82,83]) for the prediction of metabolic syndrome, leptin/ghrelin appears to have at least as good, if not a better (in men) prediction capacity. 

Early findings suggest a different influence of fat amount on health status—the classification (according to BMI) in normal-weight vs. overweight vs. obese being too large, masking the differences in relationship with body mass index [3]. Metabolically obese normal-weight patients present hyperinsulinemia, insulin resistance, dyslipidemia, and an increased risk of cardiovascular diseases [3]. Therefore, we need more accurate instruments to differentiate between MuHOB-MONW-MHOB, such as the adipokines/BMI ratio. 

The most striking observation that emerged from the analysis was the relationship between obesity, diabetes, and the adipokines/BMI ratio. The leptin/BMI ratio increased with the degree of obesity, the presence of metabolic syndrome, or diabetes. Although we did not find a decrease in the ghrelin level in obese subjects when we took into consideration the ghrelin/BMI ratio, a decrescendo trend was obvious. A positive parallel trend with the ponderal status of the leptin/ghrelin/BMI ratio was also revealed. Not achieving statistical significance was probably due to the small number of evaluated subjects. The results are consistent with (to our best knowledge) the only published study [27] that evaluated the adipokines/BMI ratio. 

From this point of view, it seems important that not only the total adipokines’ levels but also the idea that obesity and diabetes mellitus are associated with changes in adipokines’ level/kg/m^2^ (bringing a detailed look, a finesse one about a new possible involved mechanism).

Considering the small number of participants and discrepancy between the numbers of men/women due to consecutively admitted hospital patients (both of them being important limitations of the study), further research is needed to fully assess the relationship between adipokines, obesity, diabetes, and their pathophysiological involvement. Another serious limitation of the study is the incapacity to deepen the analysis according to the obesity degree. However, we should mention the fact that, even in a small sample, significant and interesting relationships involving leptin/ghrelin, leptin/BMI, ghrelin/BMI, and leptin/ghrelin/BMI ratios have been found. 

This work provides new insights into the relationship between adipokines, diabetes, and obesity, opening new research directions to identify the changes responsible for the appearance and unfavorable disease evolution. 

## 5. Conclusions

In conclusion, this study provides the backbone for future studies. There are still many unanswered questions surrounding the release, role, and prognostic value of adipokines. The results of this study suggest that obese and diabetic patients present both an alteration of total adipokines’ level, but also changes in the relationship with body mass index. These changes seem to appear even in overweight subjects offering a base for early intervention in diabetic and obese patients. 

## Figures and Tables

**Table 1:** Subjects’ characteristics.

		Normal Weight	Overweight	Obese	*p*-Value	MetS−	MetS+	p*-Value
Patients		12 (20)	31 (51.7)	17 (28.3)		17 (28.3)	43 (71.7)	
Age		64.58 ± 8.09	63.29 ± 10.79	57.41 ± 9.01	*p* = 0.08	59.88 ± 9.17	62.67 ± 10.42	*p* = 0.33
Gender	Female	10 (83.33)	22 (70.96)	12 (70.58)	*p* = 0.48	14 (82.35)	30 (69.76)	*p* = 0.50
	Male	2 (16.66)	9 (29.03)	5 (29.41)		3 (17.64)	13 (30.23)	
WC		85.16 ± 9.59	97.48 ± 7.16	107.70 ± 6.88	*p* < 0.001	91.76 ± 11.73	100.34 ± 9.5	*p* = 0.0046
BMI (kg/m^2^)		23.22 ± 1.89	27.68 ± 1.52	33.59 ± 2.35	*p* < 0.001	25.98 ± 3.99	29.44 ± 3.75	*p* = 0.0025
Systolic blood pressure		126.25 ± 17.46 (120)	133.22 ± 16.66 (130)	134.41 ± 17.84 130)	*p* = 0.40	120.58 ± 14.45	136.74 ± 16.03	*p* = 0.0006
Diastolic blood pressure *		75.41 ± 5.82 (80)	86.45 ± 20.46 (80)	84.41 ± 13.67 (80)	*p* = 0.049	77.94 ± 10.16 (80)	85.93 ± 18.62 (80)	*p* = 0.072
Diabetes	Yes	1(8.33)	5 (16.12)	8 (47.05)	*p* = 0.0099	0 (0)	14 (32.55)	*p* = 0.0189
	No	11 (91.66)	26 (83.87)	9(52.94)		17 (100)	29 (67.44)	
Hypertension	Yes	7 (58.33)	25 (80.64)	15 (88.23)	*p* = 0.06	8 (47.05)	39 (90.69)	*p* = 0.0008
	No	5(41.66)	6 (19.35)	2 (11.76)		9 (52.94)	4 (9.3)	
Current smokers	Yes	2 (16.66)	5 (16.12)	4 (23.52)	*p* = 0.60	3 (17.64)	8 (18.60)	*p* = 0.77
	No	10 (83.33)	26 (83.87)	13 (76.47)		14 (82.35)	35 (81.39)	
Glycemia * (mg/dL)		86.50 ± 6.54 (86)	101.12 ± 45.77 (91)	110.76 ± 30.31 (105)	*p* = 0.016	85.94 ± 8.09 (86)	106.86 ± 42.48 (97)	*p* = 0.005
Dyslipidemia	Yes	9 (75)	20 (64.51)	12 (70.58)	*p* = 0.86	12 (70.58)	29 (67.44)	*p* = 0.94
	No	3 (25)	11 (35.48)	5 (29.41)		5 (29.41)	14 (32.55)	
Total-C (mg/dL)		224.5 ± 51.60	207.83 ± 40.86	210.82 ± 61	*p* = 0.60	220.94 ± 41.63	208.48 ± 51.58	*p* = 0.37
LDL-C (mg/dL)		146 ± 42.13	134.67 ± 30.87	129.58 ± 47.03	*p* = 0.52	143.76 ± 35.84	132.23 ± 38.81	*p* = 0.29
Triglycerides (mg/dL)		165.08 ± 68.23	149.77 ± 67.05	171.70 ± 89.11	*p* = 0.59	122.76 ± 47.64	173.39 ± 77.37	*p* = 0.003
HDL-C (mg/dL)		45.50 ± 8.67	43.22 ± 10.42	40.82 ± 9.37	*p* = 0.44	52.70 ± 8.32	39.16 ± 7.41	*p* < 0.0001
Leptin * (pg/mL)		13,004 ± 8955 (14,350)	24,134 ± 23,769 (18,000)	39,284 ± 26,063 (34,360)	*p* = 0.0049	19,132 ± 19,904 (13,640)	28,995 ± 25,027 (21,500)	*p* = 0.11
Insulin (μU/mL) *		7.19 ± 0.28 (7.05)	7.97 ± 1.35 (7.4)	9.02 ± 3.43 (7.5)	*p* = 0.008	7.35 ± 0.41 (7.3)	8.41 ± 2.45 (7.4)	*p* = 0.08
HOMA index *		1.53 ± 0.15 (1.51)	2.06 ± 1.41 (1.73)	2.46 ± 1.07 (2.18)	*p* = 0.003	1.56 ± 0.19 (1.57)	2.27 ± 1.36 (1.83)	*p* = 0.0040
Ghrelin * (pg/mL)		37.16 ± 9.49 (36)	39.11 ± 21.81 (33)	42.02 ± 18.76 (36)	*p* = 0.70	42.91 ± 25.07 (36)	38.22 ± 15.99 (33)	*p* = 0.37
Leptin/ghrelin ratio		370.70 ± 257 (448)	771.36 ± 921 (396.46)	1055.31 ± 681.64 (985)	*p* = 0.0228	525.03 ± 584.30 (368)	869.19 ± 845 (564)	*p* = 0.0797

BMI = body mass index; Total-C = total cholesterol; LDL-C = LDL cholesterol = low-density lipoprotein; HDL-C = HDL cholesterol = high-density lipoprotein cholesterol; WC = waist circumference; HOMA index = homeostatic model assessment; * does not respect the normal distribution; data are presented as mean ± standard deviation (median value); for categorical data as number (percentage); *p* was calculated with Student’s test, Mann–Whitney test, or χ2 test; for *p* trend ANOVA (analysis of variance) or Kruskal–Wallis test were used; *p* = *p* trend normal weight vs. overweight vs. obese; p* = *p* between MetS+ vs. Mets−; NS (not statistically significant) *p* > 0.05.

**Table 2:** Relationship between adipokines and diabetes’ presence.

	DM +			DM−			*p* Global	*p* Women	*p* Men
	Global	Women	Men	Global	Women	Men			
	14 (23.3%) patients	9	5	46 (76.7%) patients	35	11			
Leptin * (pg/mL)	36,309 ± 30,848 (27,190)	51,488 ± 28,168 (36,650)	8986 ± 7366 (8500)	23,124 ± 20,867 (18,270)	27,942 ± 21,395 (22,200)	7797.27 ± 7971 (5150)	*p* = 0.13	*p* = 0.0074	*p* = 0.58
Insulin (μU/mL) *	9.2 ± 3.28 (7.75)	10.17 ± 3.79 (9.3)	7.44 ± 0.35 (7.5)	7.78 ± 1.55 (7.4)	7.93 ± 1.75 (7.4)	7.32 ± 0.40 (7.2)	*p* = 0.0253	*p* = 0.0118	*p* = 0.58
HOMA index *	3.06 ± 2.04 (2.39)	3.62 ± 2.36 (2.69)	2.04 ± 0.58 (2.12)	1.77 ± 0.53 (1.64)	1.79 ± 0.59 (1.64)	1.71 ± 0.29 (1.67)	*p* = 0.0002	*p* = 0.0002	*p* = 0.26
Ghrelin * (pg/mL)	34.14 ± 16.54 (25.7)	34.16 ± 16.92 (26)	34.10 ± 17.79 (25)	41.19 ± 19.42 (35.5)	42.67 ± 21.35 (36)	36.50 ± 10.79 (33)	*p* = 0.0409	*p* = 0.0626	*p* = 0.44
Leptin/ghrelin ratio	1224.71 ± 1114 (1035)	1742.93 ± 1072 (1409)	291.93 ± 213 (326.45)	633.80 ± 615.7 (450.99)	762.04 ± 644 (564)	225.79 ± 228 (110)	*p* = 0.0131	*p* = 0.0055	*p* = 0.58

HOMA index = homeostatic model assessment; DM = diabetes mellitus; * does not respect the normal distribution; data are presented as the mean ± standard deviation (median value); *p* was calculated with Student’s test, Mann–Whitney test; *p* global—*p* between diabetic vs. non-diabetic patients; *p* women = *p* between diabetic women vs. non-diabetic women; *p* men = *p* between diabetic men vs. non-diabetic men.

**Table 3:** The relationship between adipokines/BMI ratio and ponderal status, metabolic syndrome, and diabetes.

	Global	Normal Weight	Overweight	Obese	*p*	MetS−	MetS+	p*	DM−	DM+	*p* ^+^
L/BMI ratio *	894.15 ± 781 (710)	564.48 ± 381 (660.24)	868.03 ± 845 (618.61)	1174.49 ± 803 (1039.12)	*p* = 0.0717	687.47 ± 589 (570.82)	975.86 ± 837 (741.97)	*p* = 0.25	808.3 ± 661 (680)	1176 ± 1070 (818.96)	*p*=0.33
G/BMI ratio	1.41 ± 0.68	1.59 ± 0.35	1.41 ± 0.8	1.26 ± 0.58	*p* = 0.44	1.66 ± 0.92	1.31 ± 0.53	*p* = 0.07	1.49 ± 0.68	1.12 ± 0.57	*p* = 0.06
L/G/BMI ratio	26.47 ± 26.83	16.28 ± 11.48	27.82 ± 33.3	31.20 ± 19.47	*p* = 0.31	18.98 ± 17.84	29.43 ± 29.29	*p* = 0.09	22.47 ± 20.5	39.62 ± 39.58	*p* = 0.03

BMI = body mass index; L/BMI ratio = leptin/BMI ratio; G/BMI ratio = ghrelin/BMI ratio; L/G/BMI ratio = leptin/ghrelin/BMI ratio; DM = diabetes mellitus; * does not respect the normal distribution; data are presented as the mean ± standard deviation (median value); *p* was calculated with Student’s test, Mann–Whitney test; for *p* trend ANOVA (analysis of variance) or Kruskal–Wallis test were used; *p* = *p* trend normal weight vs. overweight vs. obese; p* = *p* between MetS+ vs. Mets−; *p*^+^ = *p* between DM+ vs. DM−.

**Table 4:** AUROCs for adipokines and the HOMA index—for metabolic syndrome identification.

	Women				Men			
	**AUROC**	Se	Sp	Criterion	**AUROC**	Se	Sp	Criterion
**L/G ratio**	**0.690**	60	78.6	>600.54	**0.923**	76.9	100	>101.98
**L**	**0.706**	83.3	57.1	>17,910	**0.821**	53.8	100	>5150
**G**	**0.536**	53.3	71.4	≤33	**0.718**	84.6	66.7	≤43
**HOMA**	**0.752**	53.33	100	1.83	**0.654**	38.46	100	1.83

L/G = leptin/ghrelin ratio, L = leptin, G = ghrelin, HOMA index = homeostatic model assessment, AUROC = area under the ROC curve; Se = sensibility, Sp = specificity.

## Abbreviations

BMI	Body mass index
WC	Waist circumference
IDF	International Diabetes Federation
HOMA index	Homeostatic model assessment index
MetS	Metabolic syndrome
HDL-cholesterol	HDL-C = high-density lipoprotein cholesterol
LDL-cholesterol	LDL-C = low-density lipoprotein cholesterol
Total-C	Total cholesterol
DM	Diabetes mellitus
AUROC	Area under the receiver operating characteristic
MuHOB	Metabolically UnHealthy Obesity
MONW	Metabolically Obese Normal Weight (MONW
MHOB	Metabolically Healthy Obesity
SD	Standard deviation

## References

[1] Atas U., Erin N., Tazegul G., Elpek G.O., Yildirim B. (2021). Changes in ghrelin, substance P and vasoactive intestinal peptide levels in the gastroduodenal mucosa of patients with morbid obesity. Neuropeptides.

[2] Cozma A., Sitar-Taut A., Urian L., Fodor A., Suharoschi R., Muresan C., Negrean V., Sampelean D., Zdrenghea D., Pop D. (2018). Unhealthy lifestyle and the risk of metabolic syndrome—The Romanian experience. JMMS.

[3] Ahima R.S., Lazar M.A. (2013). The health risk of obesity—Better metrics imperative. Science.

[4] Internation Diabetes Federation (2019). IDF Diabetes Atlas Ninth. Atlas de la Diabetes de la FID 2019.

[5] Romacho T., Elsen M., Röhrborn D., Eckel J. (2014). Adipose tissue and its role in organ crosstalk. Acta Physiol..

[6] Poher A.L., Tschöp M.H., Müller T.D. (2018). Ghrelin regulation of glucose metabolism. Peptides.

[7] Landecho M.F., Tuero C., Valentí V., Bilbao I., de la Higuera M., Frühbeck G. (2019). Relevance of leptin and other adipokines in obesity-associated cardiovascular risk. Nutrients.

[8] Frühbeck G., Toplak H., Woodward E., Yumuk V., Maislos M., Oppert J.M. (2013). Obesity: The gateway to ill health—An EASO position statement on a rising public health, clinical and scientific challenge in Europe. Obes. Facts.

[9] Fodor A., Cozma A., Suharoschi R., Sitar-Taut A., Roman G. (2019). Clinical and genetic predictors of diabetes drug’s response. Drug Metab. Rev..

[10] Chait A., den Hartigh L.J. (2020). Adipose Tissue Distribution, Inflammation and Its Metabolic Consequences, Including Diabetes and Cardiovascular Disease. Front. Cardiovasc. Med..

[11] Budak E., Fernández Sánchez M., Bellver J., Cerveró A., Simón C., Pellicer A. (2006). Interactions of the hormones leptin, ghrelin, adiponectin, resistin, and PYY3-36 with the reproductive system. Fertil. Steril..

[12] Derosa G., Catena G., Gaudio G., D’Angelo A., Maffioli P. (2020). Adipose tissue dysfunction and metabolic disorders: Is it possible to predict who will develop type 2 diabetes mellitus? Role of markers in the progression of dIabetes in obese patients (The RESISTIN trial). Cytokine.

[13] Vecchié A., Dallegri F., Carbone F., Bonaventura A., Liberale L., Portincasa P., Frühbeck G., Montecucco F. (2018). Obesity phenotypes and their paradoxical association with cardiovascular diseases. Eur. J. Intern. Med..

[14] Catoi A., Parvu A., Andreicut A., Mironiuc A., Craciun A., Catoi C., Pop I. (2018). Metabolically Healthy versus Unhealthy Morbidly Obese: Chronic Inflammation, Nitro-Oxidative Stress, and Insulin Resistance. Nutrients.

[15] Kyrou I., Mattu H.S., Chatha K., Randeva H.S. (2017). Fat Hormones, Adipokines. Endocrinology of the Heart in Health and Disease.

[16] Unamuno X., Gómez-Ambrosi J., Rodríguez A., Becerril S., Frühbeck G., Catalán V. (2018). Adipokine dysregulation and adipose tissue inflammation in human obesity. Eur. J. Clin. Investig..

[17] Adamska-Patruno E., Ostrowska L., Goscik J., Pietraszewska B., Kretowski A., Gorska M. (2018). The relationship between the leptin/ghrelin ratio and meals with various macronutrient contents in men with different nutritional status: A randomized crossover study. Nutr. J..

[18] Flehmig G., Scholz M., Kloting N., Fasshauer M., Tonjes A., Stumvoll M., Youn B.S., Bluher M. (2014). Identification of adipokine clusters related to the parameters of fat mass, insulin sensitivity and inflammation. PLoS ONE.

[19] Abd El-Kader S.M., Al-Jiffri O.H. (2018). Impact of weight reduction on insulin resistance, adhesive molecules and adipokines dysregulation among obese type 2 diabetic patients. Afr. Health Sci..

[20] Alzaim I., Hammoud S.H., Al-Koussa H., Ghazi A., Eid A.H., El-Yazbi A.F. (2020). Adipose tissue immunomodulation: A novel therapeutic approach in cardiovascular and metabolic diseases. Front. Cardiovasc. Med..

[21] Feijóo-Bandín S., Aragón-Herrera A., Moraña-Fernández S., Anido-Varela L., Tarazón E., Roselló-Lletí E., Portolés M., Moscoso I., Gualillo O., González-Juanatey J.R. (2020). Adipokines and inflammation: Focus on cardiovascular diseases. Int. J. Mol. Sci..

[22] Recinella L., Orlando G., Ferrante C., Chiavaroli A., Brunetti L., Leone S. (2020). Adipokines: New Potential Therapeutic Target for Obesity and Metabolic, Rheumatic, and Cardiovascular Diseases. Front. Physiol..

[23] Collazo P., Martínez-Sánchez N., Milbank E., Contreras C. (2020). Incendiary leptin. Nutrients.

[24] Kim W.K., Bae K.-H., Lee S.C., Oh K.-J. (2019). The Latest Insights into Adipokines in Diabetes. J. Clin. Med..

[25] Kojta I., Chacińska M., Błachnio-Zabielska A. (2020). Obesity, Bioactive Lipids, and Adipose Tissue Inflammation in Insulin Resistance. Nutrients.

[26] Arabi Y.M., Jawdat D., Al-Dorzi H.M., Tamim H., Tamimi W., Bouchama A., Sadat M., Afesh L., Abdullah M.L., Mashaqbeh W. (2020). Leptin, ghrelin, and leptin/ghrelin ratio in critically ill patients. Nutrients.

[27] Miljković M., Šaranac L., Bašić J., Ilić M., Djindjić B., Stojiljković M., Kocić G., Cvetanović G., Dimitrijević N. (2017). Evaluation of ghrelin and leptin levels in obese, lean and undernourished children. Vojnosanit. Pregl..

[28] Al-Amodi H.S., Abdelbasit N.A., Fatani S.H., Babakr A.T., Mukhtar M.M. (2018). The effect of obesity and components of metabolic syndrome on leptin levels in Saudi women. Diabetes Metab. Syndr. Clin. Res. Rev..

[29] Pico C., Palou M., Pomar C., Rodriguez A., Palou A. (2021). Leptin as a key regulator of the adipose organ. Rev. Endocr. Metab. Disord..

[30] Ouerghi N., Feki M., Bragazzi N.L., Knechtle B., Hill L., Nikolaidis P.T., Bouassida A. (2021). Ghrelin Response to Acute and Chronic Exercise: Insights and Implications from a Systematic Review of the Literature. Sport. Med..

[31] Singh P., Zhang Y., Sharma P., Covassin N., Soucek F. (2018). Statins decrease leptin expression in human white adipocytes. Physiological reports.

[32] Takahash Y., Satoh M., Tabuchi T., Nakamura M. (2012). Prospective, randomized, single-blind comparison of effects of 6 months’ treatment with atorvastatin versus pravastatin on leptin and angiogenic factors in patients with coronary artery disease. Heart Vessel..

[33] Al-Azzam S.I., Alkhateeb A.M., Alzoubi K.H., Alzayadeen R.N., Ababneh M.A., Khabour O.F. (2013). Atorvastatin treatment modulates the interaction between leptin and adiponectin, and the clinical parameters in patients with type II diabetes. Exp. Ther. Med..

[34] Szotowska M., Czerwienska B., Adamczak M., Chudek J., Wiecek A. (2012). Effect of low-dose atorvastatin on plasma concentrations of adipokines in patients with metabolic syndrome. Kidney Blood Press. Res..

[35] Sahebkar A., Giua R., Pedone C. (2016). Impact of Statin Therapy on Plasma Leptin Concentrations: A Systematic Review and Meta-Analysis of Randomized Placebo-Controlled Trials. Br. J. Clin. Pharmacol..

[36] Yorulmaz H., Ozkok E., Erguven M., Ates G., Aydın I., Tamer S. (2015). Effect of simvastatin on mitochondrial enzyme activities, ghrelin, hypoxia-inducible factor 1α in hepatic tissue during early phase of sepsis. Int. J. Clin. Exp. Med..

[37] Gruzdeva O., Uchasova E., Dyleva Y., Akbasheva O., Karetnikova V., Shilov A., Barbarash O. (2017). Effect of different doses of statins on the development of type 2 diabetes mellitus in patients with myocardial infarction. Diabetes Metab. Syndr. Obes. Targets Ther..

[38] Pothiwala P., Jain S.K., Subhashini Y. (2009). Metabolic syndrome and cancer. Metab. Syndr. Relat. Disord..

[39] Catalán V., Gómez-Ambrosi J., Rodríguez A., Frühbeck G. (2013). Adipose tissue immunity and cancer. Front. Physiol..

[40] Alexescu T.G., Cozma A., Sitar-Tăut A., Negrean V., Handru M.I., Motocu M., Tohănean N., Lencu C., Para I. (2016). Cardiac Changes in Overweight and Obese Patients. Rom. J. Intern. Med..

[41] Cooper I., Brookler K., Crofts C. (2021). Rethinking Fragility Fractures in Type 2 Diabetes: The Link between Hyperinsulinaemia and Osteofragilitas. Biomedicines.

[42] Sitar Taut A.V., Pop D., Zdrenghea D.T. (2015). NT-proBNP values in elderly heart failure patients with atrial fibrillation and diabetes. J. Diabetes Complicat..

[43] Dadarlat-Pop A., Sitar-Taut A.-V., Zdrenghea D., Caloian B., Tomoaia R., Pop D., Buzoianu A. (2020). Profile of Obesity and Comorbidities in Elderly Patients with Heart Failure. Clin. Interv. Aging.

[44] Ernst M.C., Sinal C.J. (2010). Chemerin: At the crossroads of inflammation and obesity. Trends Endocrinol. Metab..

[45] Gateva A., Assyov Y., Tsakova A., Kamenov Z. (2018). Classical (adiponectin, leptin, resistin) and new (chemerin, vaspin, omentin) adipocytokines in patients with prediabetes. Horm. Mol. Biol. Clin. Investig..

[46] Calabrò P., Golia E., Maddaloni V., Malvezzi M., Casillo B., Marotta C., Calabrò R., Golino P. (2009). Adipose tissue-mediated inflammation: The missing link between obesity and cardiovascular disease?. Intern. Emerg. Med..

[47] Reddy P., Lent-Schochet D., Ramakrishnan N., McLaughlin M., Jialal I. (2019). Metabolic syndrome is an inflammatory disorder: A conspiracy between adipose tissue and phagocytes. Clin. Chim. Acta.

[48] Weinstein A.R., Sesso H.D., Lee I.M., Cook N.R., Manson J.A.E., Buring J.E., Gaziano J.M. (2004). Relationship of physical activity vs body mass index with type 2 diabetes in women. J. Am. Med. Assoc..

[49] Minciună I.A., Orășan O.H., Minciună I., Lazar A.L., Sitar-Tăut A.V., Oltean M., Tomoaia R., Puiu M., Sitar-Tăut D.A., Pop D. (2021). Assessment of subclinical diabetic cardiomyopathy by speckle-tracking imaging. Eur. J. Clin. Investig..

[50] Capparelli R., Iannelli D. (2021). Role of epigenetics in type 2 diabetes and obesity. Biomedicines.

[51] Kim K.S., Lee J.S., Park J.H., Lee E.Y., Moon J.S., Lee S.K., Lee J.S., Kim J.H., Kim H.S. (2021). Identification of novel biomarker for early detection of diabetic nephropathy. Biomedicines.

[52] Fringu F., Sitar-Taut A., Caloian B., Zdrenghea D., Comsa D., Gusetu G., Pop D. (2020). The role of NT pro-BNP in the evaluation of diabetic patients with heart failure. Endocr. Care.

[53] Sitar-Taut D.-A., Mocean L., Sitar-Taut A.-V. (2009). Research about implementing E-PROCORD—New medical and modeling approaches in IT & C age applied on cardiovascular profile evaluation at molecular level. J. Appl. Quant. Methods.

[54] Crujeiras A.B., Díaz-Lagares A., Abete I., Goyenechea E., Amil M., Martínez J.A., Casanueva F.F. (2014). Pre-treatment circulating leptin/ghrelin ratio as a non-invasive marker to identify patients likely to regain the lost weight after an energy restriction treatment. J. Endocrinol. Investig..

[55] Perakakis N., Farr O.M., Mantzoros C.S. (2021). Leptin in Leanness and Obesity. J. Am. Coll. Cardiol..

[56] Lee M.-W., Lee M., Oh K.-J. (2019). Adipose Tissue-Derived Signatures for Obesity and Type 2 Diabetes: Adipokines, Batokines and MicroRNAs. J. Clin. Med..

[57] Friedman J.M. (2019). Leptin and the endocrine control of energy balance. Nat. Metab..

[58] Dadarlat-Pop A., Pop D., Procopciuc L., Sitar-Taut A., Zdrenghea D., Bodizs G., Tomoaia R., Gurzau D., Fringu F., Susca-Hojda S. (2021). Leptin, galectin-3 and angiotensin II type 1 receptor polymorphism in overweight and obese patients with heart failure—Role and functional interplay. Int. J. Gen. Med..

[59] Wozniak S.E., Gee L.L., Wachtel M.S., Frezza E.E. (2009). Adipose tissue: The new endocrine organ? A review article. Dig. Dis. Sci..

[60] Hajimohammadi M., Shab-Bidar S., Neyestani T.R. (2017). Consumption of vitamin D-fortified yogurt drink increased leptin and ghrelin levels but reduced leptin to ghrelin ratio in type 2 diabetes patients: A single blind randomized controlled trial. Eur. J. Nutr..

[61] Daghestani M.H., Daghestani M., Daghistani M., El-Mazny A., Bjørklund G., Chirumbolo S., Al Saggaf S.H., Warsy A. (2018). A study of ghrelin and leptin levels and their relationship to metabolic profiles in obese and lean Saudi women with polycystic ovary syndrome (PCOS). Lipids Health Dis..

[62] Sitar-Taut A.-V., Coste S.C., Tarmure S., Orasan O.H., Fodor A., Negrean V., Pop D., Zdrenghea D., Login C., Tiperciuc B. (2020). Diabetes and Obesity-Cumulative or Complementary Effects On Adipokines, Inflammation, and Insulin Resistance. J. Clin. Med..

[63] Yamada C. (2021). Relationship between orexigenic peptide ghrelin signal, gender difference and disease. Int. J. Mol. Sci..

[64] Alamri B.N., Shin K., Chappe V., Anini Y. (2016). The role of ghrelin in the regulation of glucose homeostasis. Horm. Mol. Biol. Clin. Investig..

[65] Kadoglou N.P.E., Lampropoulos S., Kapelouzou A., Gkontopoulos A., Theofilogiannakos E.K., Fotiadis G., Kottas G. (2010). Serum levels of apelin and ghrelin in patients with acute coronary syndromes and established coronary artery disease-KOZANI STUDY. Transl. Res..

[66] Lv Y., Liang T., Wang G., Li Z. (2018). Ghrelin, a gastrointestinal hormone, regulates energy balance and lipid metabolism. Biosci. Rep..

[67] Tuero C., Valenti V., Rotellar F., Landecho M.F., Cienfuegos J.A., Frühbeck G. (2020). Revisiting the Ghrelin Changes Following Bariatric and Metabolic Surgery. Obes. Surg..

[68] Rodríguez A. (2014). Novel molecular aspects of ghrelin and leptin in the control of adipobiology and the cardiovascular system. Obes. Facts.

[69] Pop D., Peter P., Dădârlat A., Sitar-Tăut A., Zdrenghea D. (2015). Serum ghrelin level is associated with cardiovascular risk score. Rom. J. Intern. Med..

[70] Poykko S. (2005). Ghrelin, Metabolic Risk Factors and Carotid Artery Atherosclerosis.

[71] Poykko S., Kellokoski E., Horkko S., Kauma H., Kesaniemi Y., Ukkola O. (2003). Low plasma ghrelin is associated with insulin resistance, hypertension and the prevalence of type 2 diabetes. Diabetes.

[72] Razzaghy-Azar M., Nourbakhsh M., Pourmoteabed A., Nourbakhsh M., Ilbeigi D., Khosravi M. (2016). An Evaluation of Acylated Ghrelin and Obestatin Levels in Childhood Obesity and Their Association with Insulin Resistance, Metabolic Syndrome, and Oxidative Stress. J. Clin. Med..

[73] Verdeş G., Duţă C.C., Popescu R., Mituleţu M., Ursoniu S., Lazăr O.F. (2017). Correlation between leptin and ghrelin expression in adipose visceral tissue and clinical-biological features in malignant obesity. Rom. J. Morphol. Embryol..

[74] Korek E., Krauss H., Gibas-Dorna M., Kupsz J., Piątek M., Piątek J. (2013). Fasting and postprandial levels of ghrelin, leptin and insulin in lean, obese and anorexic subjects. Prz. Gastroenterol..

[75] Mohamed W.S., Hassanien M., Abokhosheim K. (2014). Role of Ghrelin, Leptin and Insulin Resistance in Development of Metabolic Syndrome in Obese Patients. Endocrinol. Metab. Syndr..

[76] Dzaja A., Dalal M.A., Himmerich H., Uhr M., Pollmächer T., Schuld A. (2004). Sleep enhances nocturnal plasma ghrelin levels in healthy subjects. Am. J. Physiol. Endocrinol. Metab..

[77] Motivala S.J., Tomiyama A.J., Ziegler M., Khandrika S., Irwin M.R. (2009). Nocturnal levels of ghrelin and leptin and sleep in chronic insomnia. Psychoneuroendocrinology.

[78] Lindqvist A., Shcherbina L., Prasad R.B., Miskelly M.G., Abels M., Martínez-Lopéz J.A., Fred R.G., Nergård B.J., Hedenbro J., Groop L. (2020). Ghrelin suppresses insulin secretion in human islets and type 2 diabetes patients have diminished islet ghrelin cell number and lower plasma ghrelin levels. Mol. Cell. Endocrinol..

[79] Labayen I., Ortega F.B., Ruiz J.R., Lasa A., Simón E., Margareto J. (2011). Role of baseline leptin and ghrelin levels on body weight and fat mass changes after an energy-restricted diet intervention in obese women: Effects on energy metabolism. J. Clin. Endocrinol. Metab..

[80] Pickhardt P.J., Graffy P.M., Zea R., Lee S.J., Liu J., Sandfort V., Summers R.M. (2021). Utilizing fully automated abdominal CT-based biomarkers for opportunistic screening for metabolic syndrome in adults without symptoms. Am. J. Roentgenol..

[81] Yoon J.H., Park J.K., Oh S.S., Lee K.H., Kim S.K., Cho I.J., Kim J.K., Kang H.T., Ahn S.G., Lee J.W. (2011). The ratio of serum leptin to adiponectin provides adjunctive information to the risk of metabolic syndrome beyond the homeostasis model assessment insulin resistance: The Korean Genomic Rural Cohort Study. Clin. Chim. Acta.

[82] Cozma A., Fodor A., Orăsan O.H., Suharoschi R., Muresan C., Vulturar R., Sampelean D., Negrean V., Pop D., Sitar-Tăut A. (2019). A comparison between insulin resistance scores parameters in identifying patients with metabolic syndrome. Stud. Univ. Babes-Bolyai Chem..

[83] Blum M.R., Popat R.A., Nagy A., Cataldo N.A., McLaughlin T.L. (2021). Using metabolic markers to identify insulin resistance in premenopausal women with and without polycystic ovary syndrome. J. Endocrinol. Investig..

